# Hierarchical Multi-Stage Attention and Dynamic Expert Routing for Explainable Gastrointestinal Disease Diagnosis

**DOI:** 10.3390/diagnostics15212714

**Published:** 2025-10-27

**Authors:** Muhammad John Abbas, Hend Alshaya, Wided Bouchelligua, Nehal Hassan, Inzamam Mashood Nasir

**Affiliations:** 1 Department of Computer Science, HITEC University Taxila, Taxila 47040, Pakistan; johnabbas@ieee.org; 2 Applied College, Imam Mohammad Ibn Saud Islamic University (IMSIU), Riyadh 11432, Saudi Arabia; hialshaya@imamu.edu.sa (H.A.); wabouchelligua@imamu.edu.sa (W.B.); 3 Department of Biomedical Engineering, HITEC University Taxila, Taxila 47040, Pakistan; nehal@crair.eu; 4 Faculty of Informatics, Kaunas University of Technology, 51368 Kaunas, Lithuania

**Keywords:** gastrointestinal disease classification, hierarchical attention mechanisms, mixture of experts framework, dynamic routing, deep learning

## Abstract

**Purpose:** Gastrointestinal (GI) illness demands precise and efficient diagnostics, yet conventional approaches (e.g., endoscopy and histopathology) are time-consuming and prone to reader variability. This work presents GID-Xpert, a deep learning framework designed to improve feature learning, accuracy, and interpretability for GI disease classification. **Methods:** GID-Xpert integrates a hierarchical, multi-stage attention-driven mixture of experts with dynamic routing. The architecture couples spatial–channel attention mechanisms with specialized expert blocks; a routing module adaptively selects expert paths to enhance representation quality and reduce redundancy. The model is trained and evaluated on three benchmark datasets—WCEBleedGen, GastroEndoNet, and the King Abdulaziz University Hospital Capsule (KAUHC) dataset. Comparative experiments against state-of-the-art baselines and ablation studies (removing attention, expert blocks, and routing) are conducted to quantify the contribution of each component. **Results:** GID-Xpert achieves superior performance with 100% accuracy on WCEBleedGen, 99.98% on KAUHC, and 75.32% on GastroEndoNet. Comparative evaluations show consistent improvements over contemporary models, while ablations confirm the additive benefits of spatial–channel attention, expert specialization, and dynamic routing. The design also yields reduced computational cost and improved explanation quality via attention-driven reasoning. **Conclusion:** By unifying attention, expert specialization, and dynamic routing, GID-Xpert delivers accurate, computationally efficient, and more interpretable GI disease classification. These findings support GID-Xpert as a credible diagnostic aid and a strong foundation for future extensions toward broader GI pathologies and clinical integration.

## 1. Introduction

The global burden of gastrointestinal disease was heavy in 2019, with an age-standardized rate of 95,582 incident cases per 100,000 person-years (95% UI, 87,741–104,084 incident cases) across 204 countries and territories, which translates to 7.3 billion incident cases (95% UI, 6.7–9.0 billion) [1]. Gastrointestinal diseases are diseases of the gastrointestinal tract, i.e., the esophagus, stomach, small intestine, large intestine, and rectum; and the accessory organs of digestion, the liver, gallbladder, and pancreas [2]. The incident cases, deaths, and DALYs of digestive diseases in 2019 worldwide were 443.53 million, 2.56 million, and 88.99 million, with respective increases of 74.44, 37.85, and 23.46% compared with those in 1990 [3]. Gastrointestinal (GI) alterations in the elderly are usual, and although certain GI disorders have a greater occurrence in the elderly, no GI disease is exclusive to this population. Although specific alterations of the aging GI system are physiologic, others are pathologic and especially more common in individuals older than 65 years old [4]. Digestive diseases like colorectal cancer, gastric ulcers, and inflammatory bowel diseases have been estimated by the World Health Organization (WHO) to be responsible for a high prevalence rate of disease across the world, with an increasing incidence rate observed due to eating habits, lifestyle, and environmental factors [5]. Among GI diseases, colorectal cancer alone results in more than 1.9 million new cases annually, with gastric cancer and esophageal cancer exerting a significant influence on cancer deaths worldwide [6].

Conventionally, GI disease has been diagnosed by invasive methods such as endoscopic examination, biopsy, and histopathological examination [7]. Endoscopy remains the gold standard for the identification of gastrointestinal tract pathology; nevertheless, subjective visualization of endoscopic images by hand is inconsistent and subject to specialists’ expertise [8]. Histopathology, although most precise, is labor-intensive and subject to an expert’s opinion, leading to delay in diagnosis and treatment [9]. Radiological techniques like magnetic resonance imaging (MRI) and computed tomography (CT) scans provide additional diagnostic proof but are affected by resolution deficiencies and cost [10]. Such traditional techniques, although useful, are marred by inherent shortcomings such as operator dependence, risk of misinterpretation, and delayed detection of incipient diseases [11]. Consequently, automated and dependable diagnostic tools are increasingly needed that could avoid substantial limitations, improve accuracy, decrease workload, and enable early detection of GI diseases [12].

Despite significant advancements in deep learning architectures, several gaps remain in optimizing feature extraction and classification efficiency. Many existing models lack a structured hierarchical approach, utilizing a Mixture of Experts (MoE) framework, which leads to suboptimal feature extraction and inefficient resource utilization. Traditional classification models often struggle to direct attention toward the most clinically relevant regions, which results in the processing of large amounts of irrelevant information and a failure to capture patterns that are critical for diagnosis. The absence of dedicated mechanisms for amplifying important signals further leads to redundant representations and weakens the overall quality of extracted features. Models without residual pathways are especially prone to gradient vanishing issues, which disrupt the training process, limit the exchange of information between layers, and slow down learning efficiency. Another shortcoming is the lack of adaptive routing strategies, preventing the flexible distribution of weights across specialized modules and reducing the capacity to handle inputs of varying complexity. In addition, many designs omit intermediate auxiliary classifiers, despite their usefulness in strengthening gradient propagation, improving early-stage feature learning, and mitigating overfitting by introducing supplementary classification objectives before the final output. Parameter tuning also remains a persistent difficulty, as conventional manual or exhaustive search approaches are still widely used, whereas probabilistic optimization methods such as the Tree-Structured Parzen Estimator can offer faster convergence and better results. Addressing these limitations can pave the way for more effective and resilient classification frameworks in demanding diagnostic applications.

The key contributions of this study are highlighted as follows:

- A hierarchical multi-stage architecture incorporating a Mixture of Experts framework is employed in order to refine the feature extraction step by step, distribute learning across specialized expert blocks, and incorporate the capability to dynamically choose the best-suited expert blocks for maximizing classification efficiency.
- Each expert block is incorporated with a spatial-channel attention mechanism to increase the feature representation, concentrate on diagnostically meaningful areas, reduce irrelevant information, and adapt control attention weights for better disease classification.
- Squeeze-and-excitation blocks are incorporated into each expert block to recalibrate channel-wise feature responses, emphasizing key diagnostic patterns while reducing redundant information and retaining essential characteristics for accurate classification.
- Residual connections have been incorporated in the architecture to avoid the vanishing gradient problem, retain low-level features, carry information smoothly between different layers, and enhance converging speed and robustness for a more stabilized training procedure.
- Dynamic routing mechanism is incorporated simultaneously in order to allocate relative weights to the expert blocks, so that the feature can be explicitly carried out, and dynamically controls the contributions of each expert block in line with the complexity of input data, so as to render generalized enhancement in an efficient way to the model.
- Intermediate auxiliary classifiers are incorporated to enhance the flow of gradients, improving feature learning at earlier stages, and effectively reducing overfitting by enforcing extra classification tasks prior to the final decision process.
- For hyperparameter optimization, Tree-Structured Parzen Estimator is employed to automate parameter tuning, optimize model efficiency, and ensure faster convergence with improved performance.

The architecture of this work is as follows: Section 2 presents the related literature. The complete methodology, along with mathematical modeling, is presented in Section 3. Results of the proposed model are discussed in Section 4, whereas Section 5 concludes this paper with future directions.

## 2. Literature Review

GI diseases, encompassing inflammatory disorders and malignant conditions, represent a substantial global health burden, requiring accurate and rapid diagnostic techniques. Conventional diagnostic approaches, including endoscopy, histopathology, and radiological imaging, are widely used; however, these techniques are often laborious, subject to subjective interpretation, and require specialist involvement. Recent breakthroughs in deep learning have demonstrated significant potential in automating the classification of gastrointestinal diseases, utilizing feature extraction approaches to enhance diagnostic accuracy. Although convolutional neural networks (CNNs) are widely utilized for medical image processing, current models encounter difficulties in feature extraction, generalizability, and interpretability.

Escobar et al. [13] introduced a transfer learning-based solution for endoscopic image-based classification of gastrointestinal diseases, solving the computational complexity issue in current models. The traditional deep learning technique is accurate but involves huge parameters, which constrains its practicality. To avoid this, the authors presented a light CNN-based method using transfer learning with VGG-16 fine-tuned from the block4_conv2 layer. The model achieved 98% accuracy and required significantly fewer parameters than other approaches. Ablation experiments compared several CNN architectures, including DenseNet201, ResNet50, Xception, and VGG19, and found that VGG-16 provided the best balance between accuracy and computational cost. Baseline model benchmarking revealed improved performance, with equivalent accuracy to the state of the art, while utilizing significantly fewer computational resources. The authors demonstrated that lightweight models can achieve high classification accuracy after fine-tuning and are thus ideal for real-time clinical use.

Alhajlah et al. [14] suggested a deep learning framework for classifying gastrointestinal disease to overcome the issue of similarity between infected and normal areas. Classical approaches had limitations in correct classification because overlapping features caused misclassification. To improve diagnostic accuracy, the authors used Mask Recurrent-Convolutional Neural Network (R-CNN) for preliminary infection localization and fine-tuned pre-trained ResNet-50 and ResNet-152 models for feature extraction. The extracted features were combined using a serial method to maximize information retention. However, to minimize redundancy and enhance classification accuracy, the research work utilized an Improved Ant Colony Optimization (ACO) algorithm in feature selection to select the most significant features. Machine learning approaches were employed to conduct the last classification, achieving 96.43% accuracy on a widely available dataset. A comparative evaluation demonstrated that the envisioned method outperformed other available methods in both precision and efficiency.

Noor et al. [15] introduced a classification framework for gastrointestinal (GI) diseases that leverages deep learning while addressing the inherent difficulties of analyzing wireless capsule endoscopy (WCE) images. WCE data typically suffers from poor contrast and high intra- and inter-class similarity, which makes manual inspection not only labor-intensive but also prone to errors. To mitigate this challenge, the authors designed a brightness-controlled contrast enhancement technique optimized with a genetic algorithm (GA) that dynamically adjusts contrast and brightness levels, thereby improving visual quality. The enhancement was validated using quantitative measures such as peak signal-to-noise ratio (PSNR), mean square error (MSE), visual information fidelity (VIF), and information quality index (IQI), all of which confirmed significant improvements. When coupled with transfer learning and standard classifiers, this method achieved a classification accuracy of 96.40%, demonstrating that better image quality directly contributes to improved diagnostic performance and the potential for early detection of GI diseases.

Abraham et al. [16] developed a transfer learning approach for detecting and classifying digestive disorders, with emphasis on gastrointestinal diseases such as cancer, heartburn, irritable bowel syndrome (IBS), and lactose intolerance. The study tested endoscopic image data using several pre-trained networks—ResNet50, InceptionV3, DenseNet121, and EfficientNetB0. The results showed clear gains over conventional methods, with EfficientNetB0 providing the highest accuracy (98.01%), precision (98%), and recall (98%). Compared to earlier work, this framework achieved superior outcomes, indicating that transfer learning can considerably enhance computer-aided diagnostic tools. The authors also suggested that such models can be extended to other medical imaging domains in combination with image enhancement to boost reliability.

Khan et al. [17] proposed GestroNet, an intelligent system for the automatic detection and classification of GI tract diseases, including bleeding, polyps, and ulcers. To overcome challenges in lesion segmentation due to varying shapes and locations, the framework used deep saliency maps for region extraction, combined with Bayesian optimization for feature selection. MobileNet-V2, fine-tuned through transfer learning, served as the backbone, while a hybrid whale optimization algorithm eliminated redundant features. The system was validated on three datasets—Kvasir 1, Kvasir 2, and CUI Wah—achieving outstanding accuracies of 99.61%, 98.20%, and 98.02%, respectively. Compared to existing methods, GestroNet exhibited superior performance, underscoring the benefits of combining deep architectures with optimization algorithms.

Noor et al. [18] presented a deep learning model for GI disease classification that integrates an attention mechanism to enhance the focus on pathological regions. Their framework employed a lightweight CNN for feature extraction, with subsequent dimensionality reduction using a cosine similarity-based feature selection method. When tested on the Kvasir dataset, the approach yielded an accuracy of 97.68%. The results showed that attention-driven feature refinement and selection improve both classification accuracy and robustness, making the framework suitable for real clinical use.

Lonseko et al. [19] developed an attention-guided CNN framework for GI disease classification that incorporates an encoder–decoder structure with a spatial attention module. This mechanism selectively emphasizes diagnostically relevant regions of the image. Data augmentation and five-fold cross-validation were applied to counter dataset imbalance and ensure reliable evaluation. Compared with standard models such as ResNet50, GoogLeNet, and DenseNet, the proposed model achieved superior results, with accuracy up to 93.19%. Validation with t-SNE visualization and confusion matrices further confirmed its effectiveness. The study highlighted the contribution of spatial attention in improving diagnostic accuracy for gastrointestinal imaging tasks.

Overall, the reviewed studies reveal several recurring limitations in existing GI disease classification approaches. Many conventional architectures exhibit weak progressive feature refinement and insufficient extraction of discriminative information, resulting in moderate classification accuracy. Models frequently fail to adequately highlight clinically important regions, which increases the risk of misdiagnosis. A further weakness lies in the absence of channel-level attention, limiting the model’s ability to emphasize critical features. Training instability also arises in deeper networks due to vanishing gradients, while most systems lack adaptive mechanisms for dynamically adjusting learning weights. Additionally, insufficient early-layer learning leads to the loss of low-level diagnostic cues, and reliance on manual hyperparameter tuning remains both inefficient and resource-intensive. Addressing these shortcomings is essential for building more reliable and robust GI disease classification systems.

## 3. Methodology

In this section, we present a novel deep learning technique that can classify different gastrointestinal diseases through WCE images. Wireless Capsule Endoscopy (WCE) is a medical imaging technique used to examine the gastrointestinal tract by consuming a pill-sized capsule integrated with a camera, light source, and RF transmitter. This capsule takes thousands of images of the gastrointestinal tract and sends them to an external monitor for diagnosis. Three different WCE datasets, as discussed below, are used for training and testing of the model.

### 3.1. King Abdulaziz University Hospital Capsule (KAUHC) Dataset

**Figure 1:**
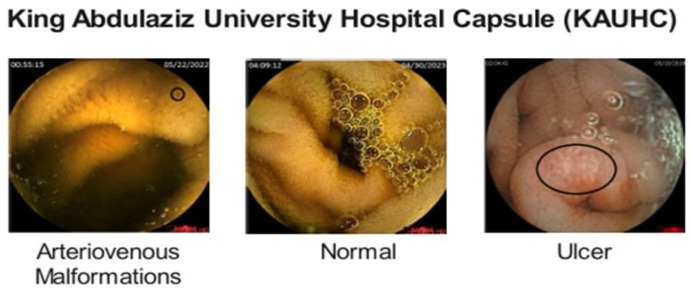
Sample Images from the KAUHC Dataset.

The KAUHC dataset (Dataset A) is a publicly available dataset comprising 3301 annotated WCE images, divided into three classes: Normal, Arteriovenous Malformations, and Ulcer. These images are collected from 86 WCE studies using OMOM capsules and recording devices. The dataset is slightly imbalanced as the number of images in the Normal class is 2156, whereas Arteriovenous Malformations and Ulcer contain 673 and 472 images, respectively. Sample images of each class are shown in Figure 1.

### 3.2. WCEBleedGen Dataset

**Figure 2:**
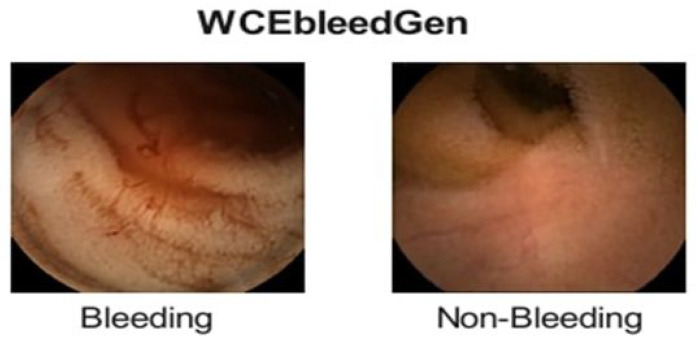
Sample Images from the WCEBleedGen Dataset.

WCEbleedGen (Dataset B) is a WCE dataset containing annotated bleeding and non-bleeding frames of GIT, collected from different open-source datasets (available at: https://zenodo.org/records/7548320, (accessed on 3 February 2025)). It is a highly balanced dataset composed of 1309 bleeding frames and 1309 non-bleeding frames. This dataset aims to train ML models for automated bleeding detection, classification, segmentation, and localization. Sample images of this dataset are shown in Figure 2.

### 3.3. GastroEndoNet Dataset

**Figure 3:**
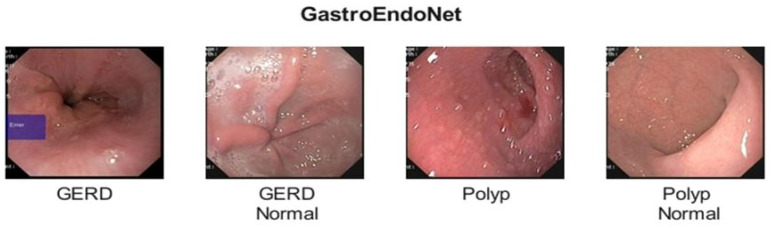
Sample Images from the GastroEndoNet Dataset.

GastroEndoNet (Dataset C) is an open-source dataset (available at: https://data.mendeley.com/datasets/ffyn828yf4/1, (accessed on 3 February 2025), which focuses on two of the most important conditions of gastroenterology, Gastroesophageal Reflux Disease (GERD) and gastrointestinal polyps. It is composed of the original 4604 images, which are augmented using six different techniques. Images are separated into four distinctive classes, such as GERD (1076), GERD Normal (1168), Polyp (1188), and Polyp Normal (1172), where GERD features images of GITs suffering from GERD, and GERD Normal contains images without GERD for comparison. Likewise, Polyp contains images of different stages and types, and Polyp Normal features images without a Polyp for comparison. Sample images of each class are shown in Figure 3.

### 3.4. Proposed GID-Xpert Model Architecture

**Figure 4:**
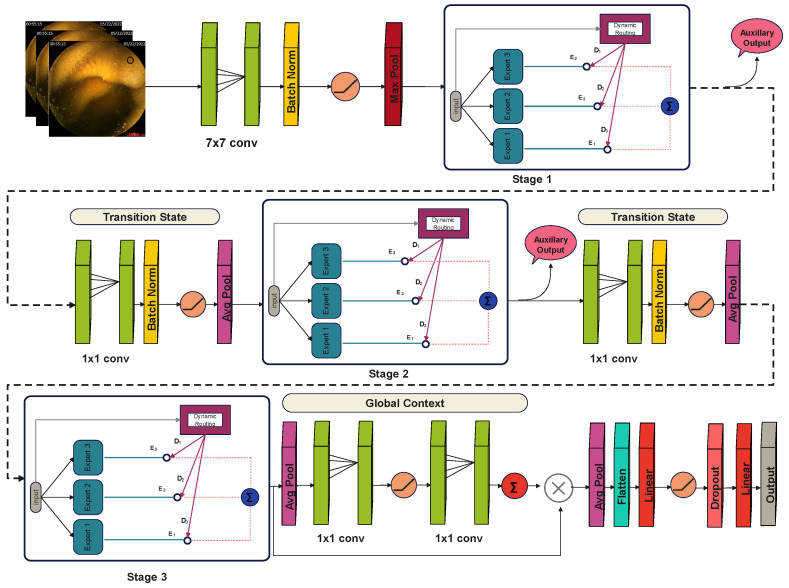
Architecture of the Proposed GID-Xpert Model for Gastrointestinal Disease Classification.

Gastrointestinal diseases contain a lot of inter-class resemblances and intra-class differences, which make it difficult for traditional models to distinguish among various classes. Moreover, these images contain both fine-grained local and global features, requiring a multi-scale feature extraction. To overcome these challenges, we proposed a hierarchical multi-stage attention-based model named “GID-Xpert”, which incorporates a mixture of experts with dynamic routing for accurate and efficient classification of different gastrointestinal diseases. The proposed approach consists of three modules: a feature extraction backbone, multi-stage expert blocks, and a dynamic routing mechanism. Additionally, the model incorporates auxiliary classifiers to facilitate efficient learning at early stages of training. A complete diagram of the proposed architecture is shown in Figure 4.

#### 3.4.1. Data Preprocessing and Feature Extraction

The model accepts the WCE images as input, resizes them to size (224, 224, 3), and applies horizontal flipping, rotation, brightness, and contrast to augment the data. The augmented data is then preprocessed using normalization techniques and enters the first module for low-level feature extraction. This module consists of a 7 × 7 convolutional layer with 64 filters, a stride of 2, and same padding, used to extract low-level features. It is followed by a batch normalization layer $\beta_{N}$ to normalize the outputs and a ReLU activation function σ to introduce non-linearity in the model. After that, a MaxPooling layer $M_{P}$ is applied with a pool size of 3 × 3 to reduce the dimensionality of the feature map. It can be defined as:

(1) $$Z^{\prime }=M_{P}\left(\sigma \left(\beta_{N}\left(W*Z+b\right)\right)\right)$$

where Z is input, b and W represent the bias term and convolutional weights, and ∗ stands for the convolutional operation. After the initial preprocessing and feature extraction, the input is then passed to a multi-stage architecture of expert blocks for enhanced feature learning.

#### 3.4.2. Multi-Stage Expert Block Architecture

The proposed module is organized into three sequential stages, with each stage containing three expert blocks. A dynamic routing mechanism regulates the relative contribution of these experts by assigning adaptive weights based on the input requirements. The input is simultaneously propagated through all experts, after which the routing mechanism computes the weights, multiplies them with the respective expert outputs, and finally concatenates the weighted results. Each expert block is designed with five main components. A spatial–channel attention unit first enhances the focus on diagnostically relevant regions of the input. This is followed by three convolutional layers that extract hierarchical features at multiple levels of abstraction. A squeeze-and-excitation unit is then applied to emphasize the most informative feature responses. Finally, a residual connection is incorporated to facilitate gradient flow, improve stability during training, and preserve information across layers.In an expert block, input is first passed through the attention block, which consists of two streams: a channel attention stream to enhance the essential channels and a spatial attention stream to focus on essential feature maps. Input is passed to both streams simultaneously. The channel attention block is composed of three layers: first, a 7 × 7 convolutional layer for feature learning; then, a batch normalization layer to stabilize the training process, followed by a sigmoid activation function to scale the features between 0 and 1. It can be defined as:

(2) $$Z_{1}=\psi \left(\beta_{N}\left(W_{1}*Z^{\prime }+b_{1}\right)\right)$$

where ψ represents the sigmoid activation function, on the other hand, the spatial attention block is comprised of 5 layers. First, an adaptive average pooling layer is used, which provides global feature understanding. Then, a 1 × 1 convolutional layer is used to reduce the number of channels by a factor of 16, thereby balancing the computational cost. A ReLU activation function then follows it to introduce non-linearity. Following that, another 1 × 1 convolutional layer is applied to restore the original number of channels. And finally, a sigmoid activation function is employed for feature scaling to highlight the most critical features and suppress the less important ones. It can be represented as:

(3) $$Z^{\prime \prime }=\frac{1}{H\times B}\sum_{m=1}^{H}\sum_{n=1}^{B}Z_{m,n,c}^{\prime }$$

And

(4) $$Z_{2}=\psi \left(W_{2}^{\prime }*\sigma \left(W_{2}*Z^{\prime \prime }\right)\right)$$

where H and B represent the height and breadth of the feature map located at (m, n) position in the c channel, and $W_{2}^{\prime }\in R^{(C/\Delta )\times C}$ and $W_{2}\in R^{C\times (C/\Delta )}$ represents the weights of convolutional layers. Here Δ represents the reduction ratio. Both attention streams generate corresponding attention weights, which are then multiplied by the original input tensor to emphasize the most important features in the feature map.

(5) $$Z_{f}=Z⊗Z_{1}⊗Z_{2}$$

where ⊗ represents element-wise multiplication. These enhanced feature maps are then passed to the 1st convolutional block, which is composed of a 1 × 1 convolutional layer to reduce the number of channels to 32. It is followed by a batch normalization layer and a ReLU activation function to stabilize the training and to enable the linear transformations. It can be represented as:

(6) $$X_{1}=\sigma \left(\beta_{N}\left(W_{x}^{1}*Z_{f}+b_{x}^{1}\right)\right)$$

This compressed feature map is then passed as input to the second convolutional block, which consists of a 3 × 3 convolutional layer with four grouped convolutions for efficient feature extraction. Similar to the first block, it is also followed by a batch normalization layer and a ReLU activation function. After feature extraction, a third convolutional block is employed, comprising a 1 × 1 convolutional layer followed by a batch normalization layer to restore the original dimensions. Mathematically, it can be represented as:

(7) $$X_{2}=\sigma \left(\beta_{N}\left(W_{x}^{2}*X_{1}+b_{x}^{2}\right)\right)$$

(8) $$X_{3}=\beta_{N}\left(W_{x}^{3}*X_{2}+b_{x}^{3}\right)$$

The output of the third convolutional block is then entered into the squeeze and excitation block, which scales the feature map to enhance the most important features. It is composed of an adaptive average pooling layer that summarizes the feature maps into a single vector, capturing global spatial information.

(9) $$X^{\prime \prime }=\frac{1}{H\times B}\sum_{m=1}^{H}\sum_{n=1}^{B}X_{3(m,n,c)}$$

It is then passed to a 1 × 1 convolutional layer, which compresses the number of channels by a factor of 16. Following that, a ReLU activation layer is added to introduce the non-linearity, which is then followed by another 1 × 1 convolutional layer to restore the original dimensions. Lastly, a sigmoid activation function is applied to scale the outputs between 0 and 1. It can be defined as:

(10) $$X_{SE}=\psi \left(W_{SE}^{2}*\sigma \left(W_{SE}^{1}*X^{\prime \prime }\right)\right)$$

These generated weights are then multiplied by the output of the third convolutional block to reinforce the effect of important features and then finally added to the original input tensor to preserve the gradient flow.

(11) $$X_{f}=\sigma \left(X_{SE}\times X_{3}\right)+Z^{\prime }$$

**Figure 5:**
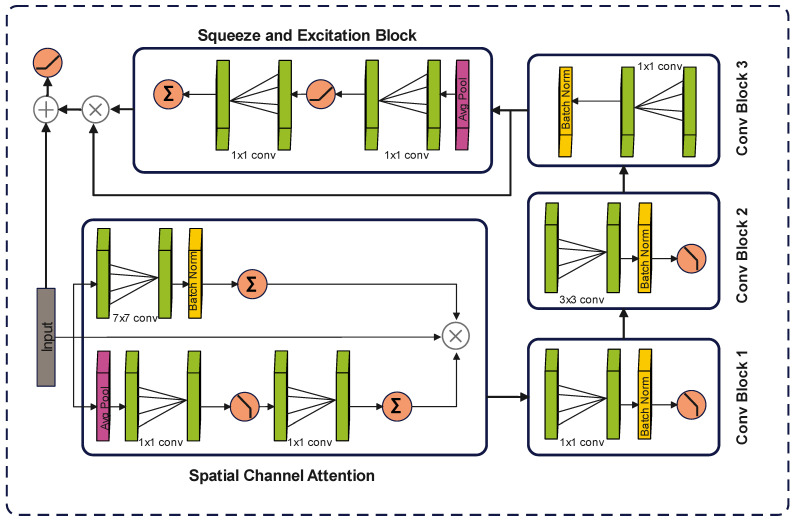
Architecture of the Proposed Expert Block in GID-Xpert.

A complete diagram of proposed Expert block is shown in the Figure 5. The outputs of expert blocks are multiplied by their corresponding weights generated by the dynamic routing mechanism.

#### 3.4.3. Dynamic Routing Module

A dynamic routing mechanism is incorporated into this architecture, which controls the influence of each expert block on the final output. It is provided with the input features and the number of experts. During the training phase, it assigns random weights and feature maps to each expert block and optimizes these weights using backpropagation. Due to their varying weights, each expert block focuses on different features in an input image, which enables them to specialize in a specific type of feature. For example, in this case, some expert block is efficient in detecting low-frequency features, while the other is good in extracting high-frequency features. During training, the Dynamic Routing System keeps track of each expert block, allowing it to determine which expert block is best suited for a specific type of feature. When a test image arrives, it examines the image and then computes the routing scores for each expert block based on the image requirements and their respective specialties. It can be defined as:

(12) $$R_{s}=Y_{GAP}W_{R}$$

Here, $Y_{GAP}$ represents the global pooled features, $W_{R}$ represents the learnable weight matrix and $R_{s}$ stands for unscaled rooting scores.

A SoftMax function is applied to these routing scores to scale them between 0 and 1 and generate final weights.

(13) $$\partial_{j}=\frac{e^{R_{j}}}{\sum_{i=1}^{N}e^{R_{i}}},\;j\in \left\{1,2,\ldots ,N\right\}$$

**Figure 6:**
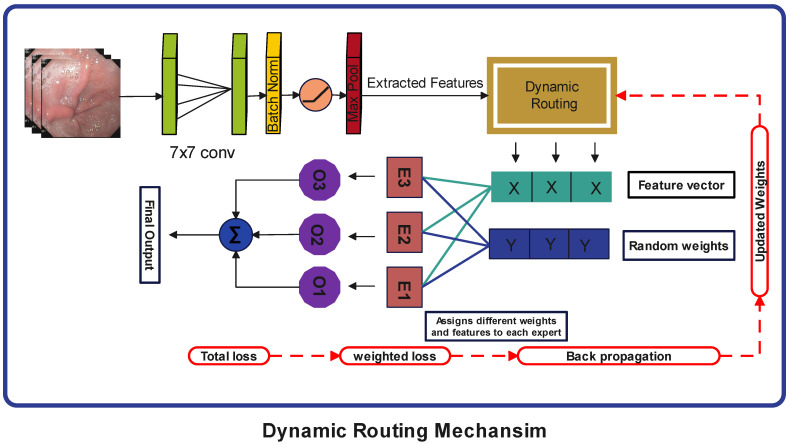
Proposed Workflow of the Dynamic Routing Mechanism.

Here, $\partial_{j}$ represents the weighted scores for each expert block. Input is passed through all the expert blocks simultaneously and then multiplied by their respective weights, which enhances the effect of the most relevant expert blocks. All the outputs are then concatenated and passed to a transition block before entering the next stage. A complete diagram of proposed flow of a dynamic routing mechanism is shown in the Figure 6.

#### 3.4.4. Feature Transition Block

A transition block is added after 1st and 2nd stages in the architecture to downsample the feature map and improve feature abstraction. Each transition block is composed of 4 layers: First, a 1 × 1 convolutional layer for channel compression and redundant feature suppression. Then, a batch normalization layer is used to stabilize training and improve convergence. After that, a ReLU activation function is used to introduce non-linearity and to capture complex patterns. And finally, a 2×2 average pooling layer to reduce the spatial dimensions for computational efficiency. Mathematically, a transition block can be defined as:

(14) $$T=A_{\rho }\left(\sigma \left(\beta_{N}\left(W_{T}*E\right)\right)\right)$$

Here, E represents the output of an expert block, and $A_{\rho }$ represents the average pooling layer. After the transition block, input is passed to the 2nd stage to extract features at higher levels. Just like the first stage, it is also composed of three expert blocks and a dynamic routing mechanism. The weighted outputs of each expert block are then concatenated and passed to another transition block for further downsampling. After that, the third and final stage of experts is incorporated in the architecture, which refines the feature maps before passing them for final classification.

#### 3.4.5. Final and Auxiliary Classifications

After passing through all the stages and transitions, the extracted feature maps are finally entered into the classification head. Just before the final classification, a global context block is incorporated to understand the global context of the entire feature map. It is comprised of an adaptive average pooling layer to summarize the spatial information, followed by two 1 × 1 convolutional layers that create a bottleneck to maintain computational efficiency. A ReLU activation function is applied between the convolutional layers to capture complex patterns. Lastly, a sigmoid activation function is added to scale the outputs between 0 and 1. It can be defined as:

(15) $$F=\psi \left(W_{G}^{2}*\left(\sigma \left(W_{G}^{1}*A_{\rho }\left(E_{3}\right)\right)\right)\right)$$

**Figure 7:**
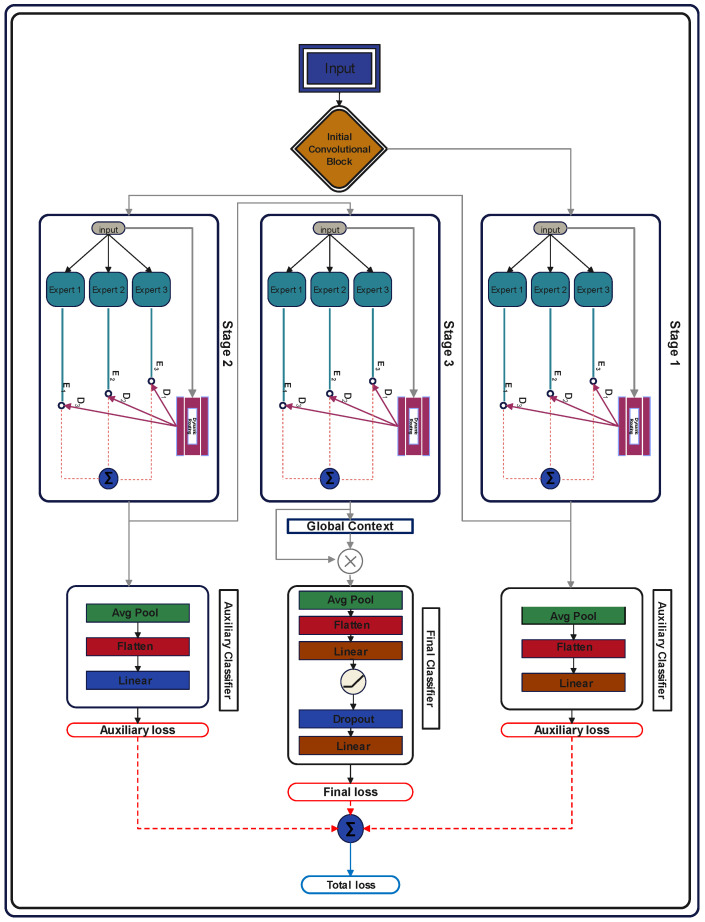
Auxiliary and Final Classification Mechanisms in the Proposed GID-Xpert Model.

These weights are then multiplied by the final output of the multi-stage architecture and fed to the final classification head for classification purposes. It is composed of an adaptive average pooling layer that summarizes the spatial information, followed by a flatten layer to convert the feature map into a 1D matrix. After that, a linear fully connected layer with 512 neurons is used to improve the feature representation. The next layer is ReLU activation, which introduces non-linearity in the model, followed by a dropout layer, which deactivates the random 50% of neurons to prevent overfitting. Finally, another dense layer is applied, which outputs the final class. Aside from the final classifier, two auxiliary classifiers are also incorporated in the architecture, one after 1st block and the other after 2nd block. These classifiers are composed of three layers: an adaptive average pool, a flatten layer, and a linear layer, which provides intermediate classification to improve learning at early stages. The outputs of these classifiers are added to the final loss during the training phase only, to improve feature learning at lower stages. The total loss can be defined as:

(16) $$L_{\mathrm{total}}=\gamma_{o}L_{\mathrm{final}}+\sum_{s=1}^{s-1}\gamma_{s}L_{\mathrm{aux},s}$$

where s represents the current stage, the proposed framework of auxiliary classifiers and final classifier is shown in the Figure 7.

### 3.5. Hyperparameter Optimization Using TPE

Unlike the model parameters, which are dynamically optimized during the training process, the hyperparameters of the model are fixed before training. Selection of optimal hyperparameters is essential because good hyperparameters can increase the performance of the model, whereas poor ones can lead to overfitting and poor generalization. To select the optimal hyperparameters, different optimization techniques are used. In this paper, we selected a Bayesian optimization technique known as “Tree-Structured Parzen Estimator (TPE)” for optimization of the model’s hyperparameters. Unlike the traditional BO, which approximates a Gaussian process over the objective function, it models two probability distributions: one for good-performing hyperparameters and the other for bad-performing hyperparameters. It works by evaluating the model’s performance on different hyperparameters and then dividing those hyperparameters into two sets and modeling their probability distribution, which can be represented as:

(17) $$g\left(z\right)=P\left(z|x<x^{*}\right)\;\mathrm{For}\;\mathrm{top}\;\mathrm{performing}$$

(18) $$b\left(z\right)=P\left(z|x>x^{*}\right)\;\mathrm{For}\;\mathrm{poor}\;\mathrm{performing}$$

Here, z is the hyperparameter configuration, x is the corresponding objective function value, and $x^{*}$ is the threshold for separating good hyperparameters from bad ones, which is the top α percentile of evaluations. Now, the next hyperparameter configuration will be evaluated by maximizing the expected improvement.

(19) $$z^{*}=\arg\max_{z}\frac{g(z)}{b(z)}$$

After all the configurations, the hyperparameter configuration with the minimum f(z) will be selected. Here, f(z) is the validation loss. The algorithm of Tree-Structured Parzen Estimator (TPE) is shown in the Algorithm 1.
**Algorithm 1** Tree-Structured Parzen Estimator (TPE) for Hyperparameter Optimization**Require:** Search space S (tree-structured), objective f:S→R to minimize, stopping criterion STOP(·), quantile γ∈(0,1), initial random trials $n_{0}$, candidates per iteration *T***Ensure:** Best configuration $x^{*}$  1:Initialize dataset D←⌀  2:**for** i=1 to $n_{0}$ **do**  3:   Sample $x_{i}∼\mathrm{Prior}\left(S\right)$ (respect tree dependencies)  4:   Evaluate $y_{i}\leftarrow f\left(x_{i}\right)$  5:   $D\leftarrow D\cup \{\left(x_{i},y_{i}\right)\}$  6:**end for**  7:$\left(x^{*},y^{*}\right)\leftarrow \arg\min_{(x,y)\in D}y$  8:**while** not STOP(D) **do**  9:   Compute threshold $y^{*}$ as the γ-quantile of {y:(x,y)∈D}10:   $D_{\ell }\leftarrow \left\{\left(x,y\right)\in D|y\leq y^{*}\right\}$    (*good set*)11:   $D_{g}\leftarrow \left\{\left(x,y\right)\in D|y>y^{*}\right\}$    (*bad set*)12:   Fit Parzen densities $l\left(x\right)\approx p\left(x|y\leq y^{*}\right)$ using $D_{\ell }$ and $g\left(x\right)\approx p\left(x|y>y^{*}\right)$ using $D_{g}$13:   **for** t=1 to *T* **do**14:       Sample candidate $x_{t}∼l\left(x\right)$ (respect conditional structure)15:       Score $s_{t}\leftarrow \frac{l(x_{t})}{g(x_{t})+\epsilon }$    (*maximize* l/g)16:   **end for**17:   $x_{\mathrm{new}}\leftarrow \arg\max_{t\in \{1,\ldots ,T\}}s_{t}$18:   $y_{\mathrm{new}}\leftarrow f\left(x_{\mathrm{new}}\right)$19:   $D\leftarrow D\cup \{\left(x_{\mathrm{new}},y_{\mathrm{new}}\right)\}$20:   **if** $y_{\mathrm{new}}<y^{*}$ **then**21:     $\left(x^{*},y^{*}\right)\leftarrow \left(x_{\mathrm{new}},y_{\mathrm{new}}\right)$22:   **end if**23:**end while**24:**return** $x^{*}$

## 4. Experimental Results

### 4.1. Experimental Setup and Training Configuration

This research presents a novel deep learning approach that incorporates a Mixture of Experts along with a dynamic routing mechanism. The proposed model is trained and evaluated on three datasets: the KAUHC dataset, the GastroEndoNet dataset, and the WCEBleedGen dataset, which are discussed in detail in Section 3. Eighty percent of the data is used for training, while the remaining 20% is used for validation. A tree-structured Parzen Estimator is applied as an optimization technique, and the suggested hyperparameters are used to train the model, as shown in Algorithm 1. Results of the proposed model on each dataset are shown in Section 4. Ablation studies and comparative analyses with pre-trained models are also performed to discuss the model’s efficiency. All experiments are conducted on a personal desktop computer with a 20 GB GPU (Nvidia RTX A4500) and 64 GB RAM, using Python 3.8 and TensorFlow 2.8.0.

### 4.2. Classification Results: KAUHC Dataset

**Figure 8:**
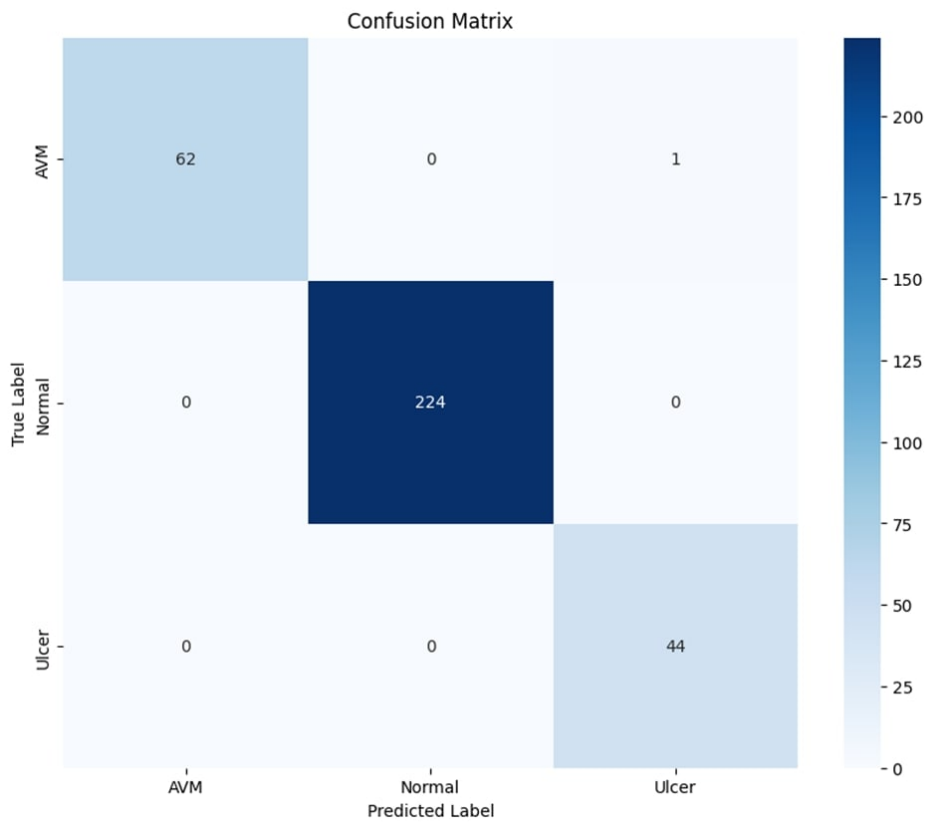
Confusion Matrix for the KAUHC Dataset.

With a test accuracy of 99.98%, the classification model demonstrated excellent performance. Table 1 shows that the metrics of precision, recall, and F1-score are consistently high among different classes, with Normal achieving perfect scores of 100%. The AVM class scores slightly lower at 98% recall, whereas the Ulcer class achieves a perfect 100% recall. All macro and weighted averages for precision, recall, and F1-score are at or above 99%, indicating an overall strong model that can be relied upon. These outcomes indicate that the model can differentiate effectively among classes AVM, Normal, and Ulcer with few slightly lower scores, thus forming a highly accurate classification system. These results can be validated through the confusion matrix shown in Figure 8.

#### 4.2.1. Stage Expert Utilization

**Figure 9:**
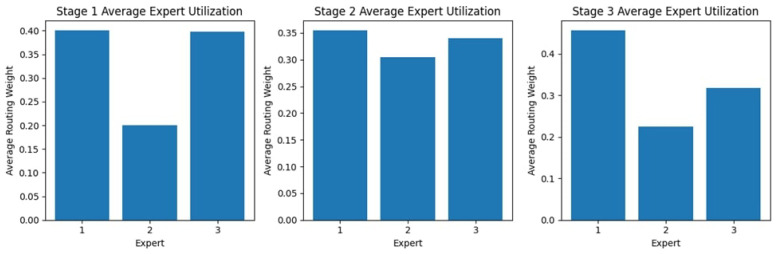
Expert Block Utilization Across Stages for the KAUHC Dataset.

Figure 9 shows the expert usage across the three stages, showing different patterns indicating a varying dependence on different experts through the process. In the first stage, Experts 1 and 3 have the most significant usage; Expert 2, on the other hand, falls below the norm, receiving a much lower routing weight. Stage 2 is more evenly utilized, with nearly equal use of Experts 1 and 3 and a slight improvement from Stage 1 for Expert 2. In Stage 3, Expert 1’s usage is at a maximum, while Expert 2 again has the lowest routing weight, and Expert 3 has moderate usage. These findings suggest possible preferences or specializations among experts, as some experts receive higher reliance at different stages.

#### 4.2.2. Routing Patterns

**Figure 10:**
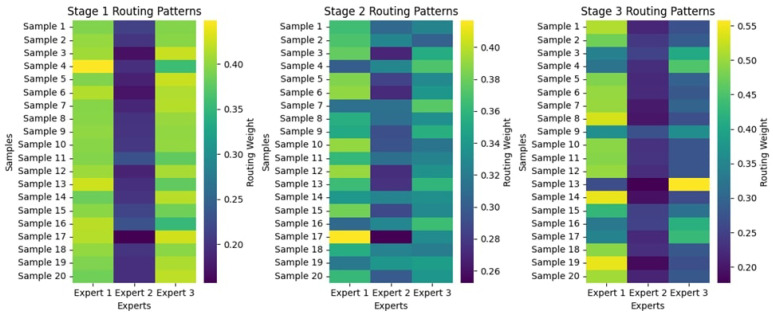
Routing Patterns Across Stages for the KAUHC Dataset.

Figure 10 illustrates the routing patterns across the three stages for 20 samples, which exhibit dynamic changes in how samples are assigned to experts based on the routing weights. Stage 1 has an uneven distribution, with some samples receiving a significantly higher weight for sure experts, most notably Experts 1 and 3. At the same time, Expert 2 is clearly underutilized due to the much lower intensities of its assigned routing weights. In Stage 2, routing weights have become more evenly distributed among the three experts, although some variation remains. By Stage 3, the routing weights for specific samples have evolved towards not only Expert 1 but also some other expert, with Expert 2 continually having lower assignments for so many. The color variations suggest that certain samples may preferentially select specific experts, potentially indicating underlying decision-making dynamics. These results suggest significant differences in how various experts are used.

#### 4.2.3. Attention Maps

**Figure 11:**
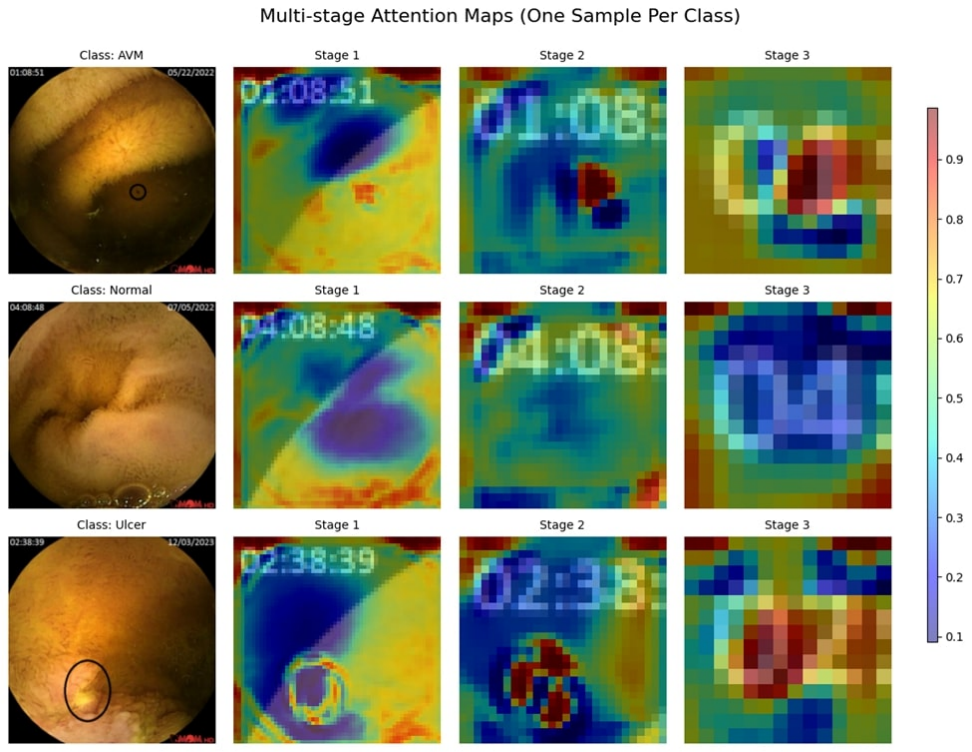
Multi-Stage Attention Maps for the KAUHC Dataset.

The multi-stage attention maps represent the sequence of feature focus across three stages for AVM, Normal, and Ulcer, as shown in Figure 11. Attention begins widely dispersed and less intense in Stage 1, considering broader areas on the map. As the stages progress, the attention map focuses on increasingly precise and smaller regions of interest. For both AVM and Ulcer, Stage 3 points almost precisely at the localized abnormality, showcasing the model’s pertinent feature focus for clear pathologies. Attention for the Normal class remains relatively dispersed, hence consistent with no evident distinguishing pathological features. Gradual progress shows that the model could filter out unimportant details and instead highlight important diagnostic clues, which justifies its good performance in classification.

### 4.3. Classification Results: WCEBleedGen Dataset

**Figure 12:**
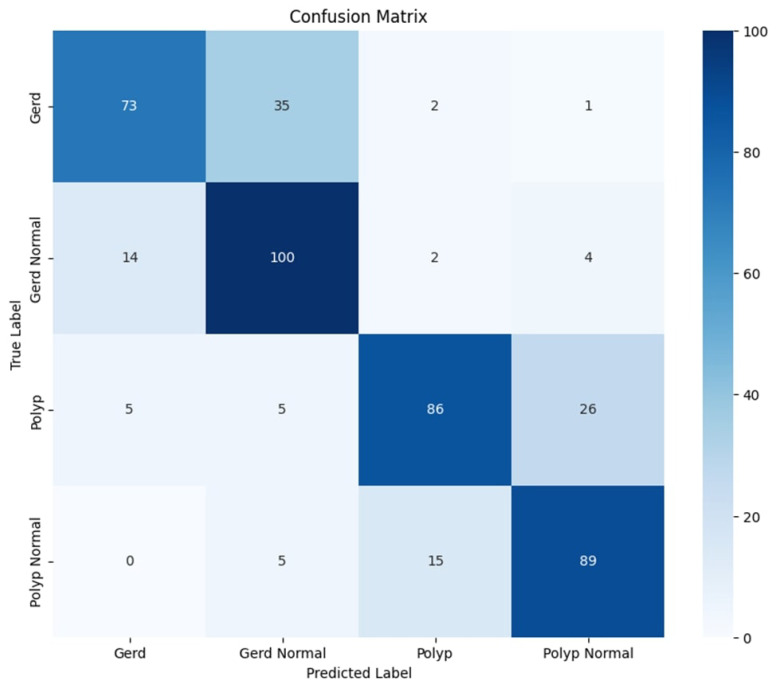
Confusion Matrix for the GastroEndoNet Dataset.

The classifying model achieves a global accuracy of 75.32%, indicating strong performance. In the context of F1-scores of classes, the range varies from 72–78%, with Polyp Normal getting the highest of 78% and Polyp getting very close to that with 76%. In terms of recall, the model performs better in identifying Gerd Normal and Polyp Normal cases, which have recall values of 83% and 82%, respectively. Gerd has the lowest recall, at 66%, thus making it a class for which there are more false negatives. For precision, the Polyp and Gerd Class is showing a precision score of 82% and 79% respectively, while the other two classes, i.e., show slightly lower values. The macro and weighted averages for precision, recall, and F1-score, however, remain stable at around 75% and reflect balanced performance across the dataset. The detailed classification report is shown in the Table 2. These results can be validated through the confusion matrix given in the Figure 12.

#### 4.3.1. Stage Expert Utilization

**Figure 13:**
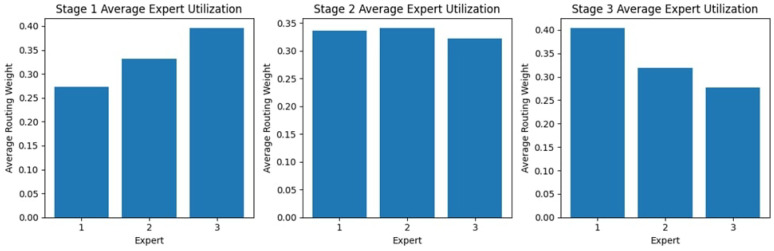
Expert Block Utilization Across Stages for the GastroEndoNet Dataset.

Figure 13 shows expert usage during the three different stages representing different distributions, indicating the extent to which a given expert was relied upon from one stage of the process to the next. In Stage 1, utility is highest for Expert 3 and lowest for Expert 1. By Stage 2, the distribution resembles a more balanced distribution, where almost similar routing weights are offered to Experts 1, 2, and 3. The next stage in this utilization pattern sees an increase in support for Expert 1, as the others recede into less routable positions. This change in utilization portrays a dynamic adaptation in the selection of experts, whereby one expert after another is increasingly relied upon for aiding in the various stages of processing. They are optimizing this process by capitalizing on the strengths of each expert.

#### 4.3.2. Routing Patterns

**Figure 14:**
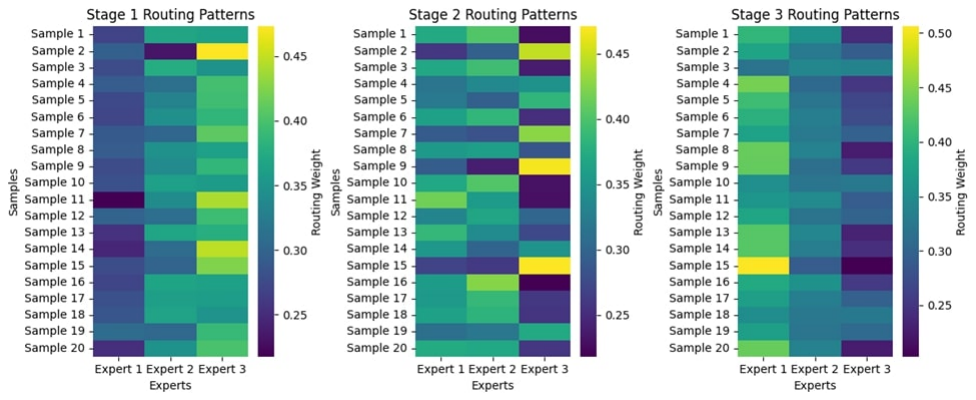
Routing Patterns Across Stages for the GastroEndoNet Dataset.

The routing patterns across the three stages, as shown in Figure 14, showcase how various samples get directed among the experts corresponding to their routing weights. In Stage 1, the distribution of these weights is more chaotic: there may be some samples that are much more reliant on one expert, while others are more equally distributed between experts. As the stages progress, routing patterns become more stable: extreme values are fewer, and weights seem to be distributed more evenly across the experts. At Stage 3, this distribution is uniform, indicating that over time, the model becomes more confident in assigning its experts. In this regard, the existence of some samples with one routing weight heavily dominating others in specific experts suggests that these experts may consistently handle some instances due to their specialization in feature extraction.

#### 4.3.3. Attention Maps

**Figure 15:**
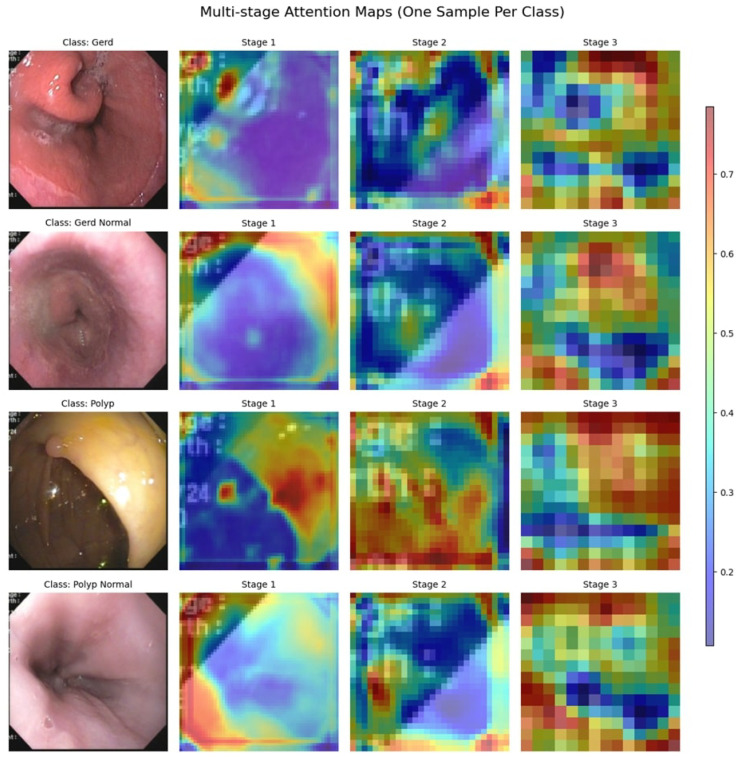
Multi-Stage Attention Maps for the GastroEndoNet Dataset.

The attention maps of the three stages, as shown in Figure 15, display a progressive enhancement in how features are extracted for different gastrointestinal conditions. In Stage 1, attention is widely distributed, concentrating on broader areas with less specificity. When going to Stage 2, attention narrows in on critical areas, with some noise remaining. Stage 3 focused its attention firmly and carefully on abnormal cases, such as GERD and Polyps. In contrast, the attention is dampened for typical cases (GERD Normal and Polyp Normal), which is in line with the absence of distinctly identifiable lesions. Such refinements in stages imply a learned ability by the model to highlight the most important diagnostic features, ultimately aiding in better classification efficiency.

### 4.4. Classification Results: GastroEndoNet Dataset

**Figure 16:**
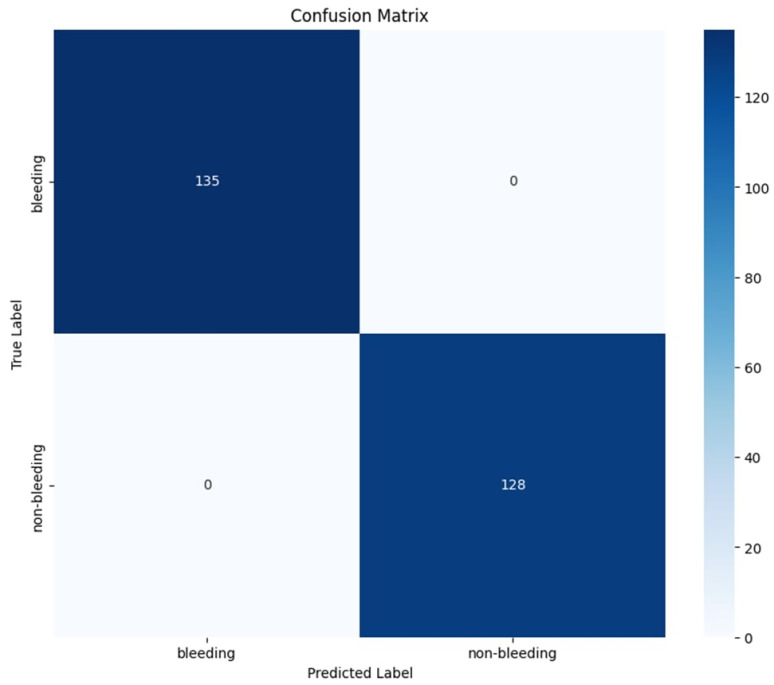
Confusion Matrix for the WCEBleedGen Dataset.

Table 3 demonstrates that the classification model is able to reach perfect accuracy: 100%, which attests to its highly qualitative performance between bleeding and non-bleeding cases. Both classes achieved a precision, recall, and F1-score of 100%, which means that none of the samples are misclassified, and each one is identified correctly. The macro and weighted averages indicate balanced performance across the dataset. These results indicate that the model has learned the distinguishing features of each class with high confidence, making it a reliable classifier for the task at hand. These results can be validated through the confusion matrix shown in the Figure 16.

#### 4.4.1. Stage Expert Utilization

**Figure 17:**
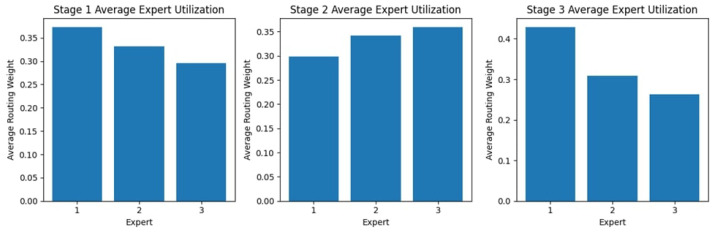
Expert Block Utilization Across Stages for the WCEBleedGen Dataset.

The expert utilization analysis across the three stages, as shown in Figure 17, reveals a dynamic distribution of routing weights. In Stage 1, Expert 1 is utilized the most, while Expert 3 has the lowest allocation. In Stage 2, the weights are more evenly distributed among Experts 2 and 3, suggesting a balanced reliance on multiple experts at this stage. However, in Stage 3, Expert 1 receives the highest routing weight, indicating that it plays a more dominant role in the final decision-making stage. This variation in expert utilization across stages highlights the model’s adaptability in leveraging different experts, depending on the complexity and nature of the classification task at each stage.

#### 4.4.2. Routing Patterns

**Figure 18:**
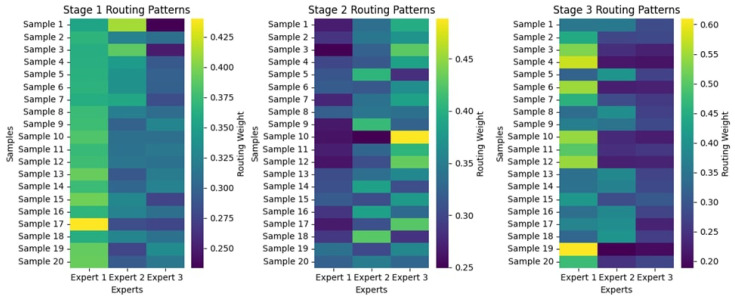
Routing Patterns Across Stages for the WCEBleedGen Dataset.

Figure 18 shows the routing configuration through three stages of the model’s decision-making process, with three experts sharing the routing weights among 20 samples. Stage 1 shows that the weighting is shared somewhat equally among experts, although specific samples display an inclination towards confident expert choices. Differentiation among experts is more easily distinguishable in Stage 2, and routing decisions have obviously been hinted at improving. By Stage 3, samples have higher confidence routed through a dominant expert in a tight concentration of weights. This is indicative of a shift from distributed to selective use of experts: given more information to process, the model relies more on specialized pathways. In this way, a deeper understanding of model interpretability and efficiency becomes clear through the lens of the hierarchical routing mechanism.

#### 4.4.3. Attention Maps

**Figure 19:**
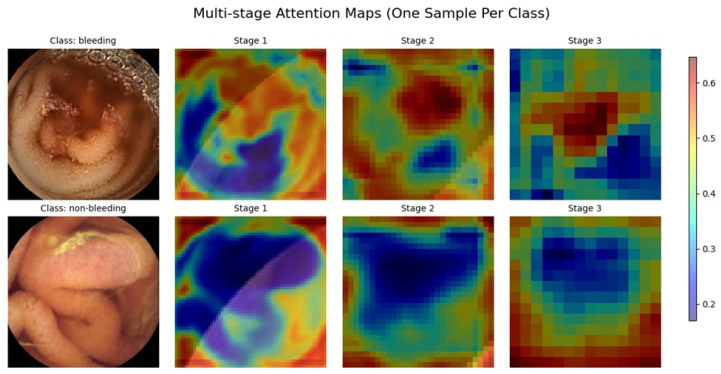
Multi-Stage Attention Maps for the WCEBleedGen Dataset.

Figure 19 shows the multi-stage attention maps for two images, one belonging to the bleeding class, the other to the non-bleeding class. The attention maps evolve in three stages, revealing how the model focuses through time and makes up its mind. In Stage 1, for the bleeding sample, attention focuses on broad features, while in the final stage, attention is more focused on the bleeding region. In non-bleeding samples, attention is dispersed in Stage 1. By the time it arrives at Stage 3, attention has mainly settled in key distinguishing areas. This sort of gradual refinement suggests the model focuses on the most critical features step by step to improve its classification precision. These findings demonstrate the effectiveness of the multi-stage attention mechanism in enhancing the interpretability of the model and its decision-making processes.

### 4.5. Ablation Studies and Analysis

#### 4.5.1. Effect of SE Blocks

In terms of performance metrics for the various architectures, the impact of the Squeeze-and-Excitation (SE) block is clear, as shown in Table 4. For the KAUHC dataset, the model without an SE block achieves an accuracy of 96.50%, with a precision of 95%, a recall of 94%, and an F1-score of 94%. However, when the SE block is added, performance goes up incredibly to 99.98% accuracy, with all classification metrics at 99%, showing improvements in feature extraction and decision-making. Similarly, in GastroEndoNet, it performed well with the addition of the SE block. It increased from an accuracy of 70.12% to 75.32%, with precision, recall, and F1-score also showing marginal gains, indicating the SE block’s role in refining predictions. For the WCE-BleedGen dataset, the model achieved an accuracy of 100% when the SE block was added, improving from 96.24%. This resulted in perfect precision, recall, and F1-score, showcasing its superior capability in classifying bleeding and non-bleeding cases.

#### 4.5.2. Effect of Spatial-Channel Attention

The Spatial Channel Attention Block (SCAB) significantly increases model performance as shown in the Table 5. In the case of KAUHC, the use of SCAB increases accuracy from 97.80% to 99.98%, with parallel gains of precision, recall, and F1-score also increasing to 99%. Similarly, with SCAB, GastroEndoNet achieved improved performance, with its accuracy increasing from 71.23 to 75.32, accompanied by modest gains in precision, recall, and F1-score, indicating good performance after the addition of SCAB. The architecture on the WCEBleedGen dataset shows the most significant improvement, achieving a complete 100% for each metric—accuracy, precision, recall, and F1-score—with SCAB included, compared to 97.50% without it.

#### 4.5.3. Effect of Number of Expert Blocks

The Ablation study 3, which examines the integration of expert blocks across different architectures, found that performance improved significantly with an increasing number of expert blocks, as shown in Table 6. On the KAUHC dataset, the accuracy increased from 97.22% with one expert block to 99.98% with three expert blocks, exhibiting similar boosts in precision, recall, and F1-score. Therefore, the addition of expert blocks improves the model’s feature extraction and processing capabilities. Similarly, accuracy on the GastroEndoNet dataset increased from 70.07% to 75.32% by increasing the number of expert modules, thus proving the advantage of adding expert modules. For the WCE-BleedGen dataset, the enhancement is the biggest, attaining 100% accuracy, precision, recall, and F1-score with three expert modules, compared to 96.24% with only one expert module. These findings highlight the effective contribution of expert blocks to tuning performance on class prediction.

#### 4.5.4. Computational Efficiency Analysis

To substantiate the claim of reduced computational cost, we analyzed the total number of trainable parameters and the average inference time per image for the proposed GID-Xpert model compared with several baseline architectures. Experiments were conducted on an NVIDIA RTX A4500 GPU using identical input dimensions and batch sizes. As shown in Table 7, GID-Xpert achieves superior computational efficiency, requiring significantly fewer parameters and lower inference time than most deep models while maintaining state-of-the-art accuracy.

These results confirm that the hierarchical mixture of experts with dynamic routing not only enhances classification accuracy but also improves computational efficiency, making GID-Xpert suitable for real-time or resource-constrained diagnostic applications.

#### 4.5.5. Comparative Evaluation with Pretrained Models

The comparison of various deep learning models, as shown in Table 8, indicates the superiority of the proposed model on all three datasets: KAUHC, GastroEndoNet, and WCE-BleedGen. In KAUHC, accuracy progresses from 94.50% with VGG-19 to 99.98% with the proposed model, indicating an increase in performance. Likewise, in GastroEndoNet, accuracy increases from 65.03% with VGG-19 and 68.50% with ResNet101 to 75.32% for the proposed model. WCE-BleedGen also shows the same trend: the proposed model attains 100% accuracy, beating VGG-19 (92.32%) and Xception (99.33%). The proposed model consistently performed the best across all datasets in terms of precision, recall, and F1-score, thereby demonstrating its superiority in handling complex medical imaging tasks with enhanced accuracy and reliability.

## 5. Conclusions and Future Work

This study introduces GID-Xpert, a hierarchical, multi-stage, attention-driven model that integrates expert blocks with dynamic routing for the classification of gastrointestinal diseases. Our approach addresses key challenges in traditional and deep learning-based GI disease diagnostics by incorporating spatial-channel attention, expert modules, and dynamic routing mechanisms. Experimental evaluations on multiple datasets demonstrate that GID-Xpert achieves superior classification performance, particularly excelling in bleeding detection with 100% accuracy on WCEBleedGen and near-perfect results on KAUHC. The model also demonstrates good performance on the GastroEndoNet dataset, pointing to its suitability for varied GI conditions. The efficacy of expert modules and attention mechanisms is confirmed through ablation studies, which stress their importance in optimizing feature extraction and classification accuracy. Although the model exhibits strong performance, its generalizability on unseen datasets and in real-life clinical settings requires further exploration. Subsequent studies may explore the integration of multimodal clinical data, optimization of computational efficiency, and the incorporation of explainable AI methods to enhance the interpretability of results. In total, GID-Xpert is a remarkable contribution to computerized GI disease diagnosis, providing a scalable and effective solution for image analysis in the medical field.

## Figures and Tables

**Table 1:** Classification Report for the KAUHC Dataset.

Class	Precision (%)	Recall (%)	F1-Score (%)	Support
AVM	100	98	99	63
Normal	100	100	100	224
Ulcer	98	100	99	44
Accuracy			99	331
Macro Avg	99	99	99	331
Weighted Avg	100	100	100	331

**Table 2:** Classification Report for the GastroEndoNet Dataset.

Class	Precision (%)	Recall (%)	F1-Score (%)	Support
Gerd	79	66	72	111
Gerd Normal	69	83	75	120
Polyp	82	70	76	122
Polyp Normal	74	82	78	109
Accuracy			75	462
Macro Avg	76	75	75	462
Weighted Avg	76	75	75	462

**Table 3:** Classification Report for the WCEBleedGen Dataset.

Class	Precision (%)	Recall (%)	F1-Score (%)	Support
bleeding	100	100	100	135
non-bleeding	100	100	100	128
Accuracy			100	263
Macro Avg	100	100	100	263
Weighted Avg	100	100	100	263

**Table 4:** Ablation Study 1: Impact of SE Blocks.

KAUHC				
Architecture	Accuracy	Precision	Recall	F1-Score
Without SE Block	96.50	95	94	94
With SE Block	99.98	99	99	99
GastroEndoNet				
Without SE Block	70.12	69	68	68
With SE Block	75.32	76	75	75
WCE-BleedGen				
Without SE Block	96.24	95	94	94
With SE Block	100	100	100	100

**Table 5:** Ablation Study 2: Impact of the Spatial-Channel Attention Block.

KAUHC				
Architecture	Accuracy	Precision	Recall	F1-Score
Without Spatial Channel Attention Block	97.80	96	95	95
With Spatial Channel Attention Block	99.98	99	99	99
GastroEndoNet				
Without Spatial Channel Attention Block	71.23	70	69	69
With Spatial Channel Attention Block	75.32	76	75	75
WCE-BleedGen				
Without Spatial Channel Attention Block	97.50	97	96	96
With Spatial Channel Attention Block	100	100	100	100

**Table 6:** Ablation Study 3: Impact of the Number of Expert Blocks.

KAUHC				
Architecture	Accuracy	Precision	Recall	F1-Score
With 1 Expert Blocks	97.22	95	94	94
With 2 Expert Blocks	98.45	97	96	97
With 3 Expert Blocks	99.98	99	99	99
GastroEndoNet				
With 1 Expert Block	70.07	69	68	68
With 2 Expert Blocks	72.50	72	71	71
With 3 Expert Blocks	75.32	76	75	75
WCE-BleedGen				
With 1 Expert Block	96.24	95	94	94
With 2 Expert Blocks	98.23	97	97	97
With 3 Expert Blocks	100	100	100	100

**Table 7:** Computational efficiency comparison across models.

Model	Parameters (Millions)	Inference Time (ms/image)
VGG-19	143.7	12.8
ResNet101	44.6	10.3
DenseNet169	14.1	9.1
InceptionV3	23.8	8.5
Xception	22.9	8.3
Proposed GID-Xpert	11.6	6.7

**Table 8:** Comparative Evaluation with Pretrained Models.

KAUHC				
Models	Accuracy	Precision	Recall	F1-Score
VGG-19	94.50	92	91	91
Resnet101	96.20	94	93	93
Densenet169	97.01	95	95	95
Inceptionv3	97.80	96	96	96
Xception	98.50	98	97	97
Proposed	99.98	99	99	99
GastroEndoNet				
VGG-19	65.03	63	62	62
Resnet101	68.50	67	66	66
Densenet169	70.50	70	69	69
Inceptionv3	72.13	72	71	71
Xception	73.32	72	71	71
Proposed	75.32	76	75	75
WCE-BleedGen				
VGG-19	92.32	91	90	90
Resnet101	95.46	94	93	93
Densenet169	97.02	96	96	95
Inceptionv3	98.23	98	97	97
Xception	99.33	98	97	97
Proposed	100	100	100	100

## Data Availability

The implementation of this work can be found at https://github.com/imashoodnasir/Gastrointestinal-Disease-Classification (accessed on 19 March 2025).
